# Sea Urchin Spine Embedded in the Sole of the Foot: Eight-Year Radiographic Follow-Up Without Removal

**DOI:** 10.7759/cureus.56261

**Published:** 2024-03-16

**Authors:** Angelina J Skedros, John G Skedros, Brett W Richards, John T Cronin

**Affiliations:** 1 Orthopaedic Surgery, Utah Orthopaedic Specialists, Salt Lake City, USA

**Keywords:** sea urchin, long follow-up, dissolve, foot, puncture

## Abstract

When sea urchin puncture injuries occur during coastal recreation or work activities, they often affect extremities, such as hands and feet. There is a plethora of information on treatments for these puncture injuries, with the most common among medical professionals being the removal of all partially embedded spines and the removal of as many fully embedded spines as possible. When the spines are deeply embedded and/or fragmented, they might not be removed, especially when they are not located in critical areas such as tendons or joints. This reflects the generally held notion that smaller spines and spine fragments will eventually dissolve or be absorbed. Here we report an unusual case where the tip of a sea urchin spine became embedded in the soft tissue of the sole of the foot of a 21-year-old male after he stepped on one after falling off a kayak off the coast of Oahu, Hawai’i. The deeply embedded spine was not removed. By three weeks after the injury, the patient did not have any symptoms, and eight years later, he was still symptom-free. Radiographs taken one year after the injury showed that the spine had fragmented into two pieces. The smaller piece was about 15% of the size of the original embedded spine, and it had apparently been absorbed (it was not seen on final radiographs eight years later). Analysis of radiographs eight years after the injury showed that the main or large spine fragment was still distinctly detectable in the soft tissue; there was no visible evidence that it had undergone significant absorption or migrated from the original location. The absence of any obvious radiographic rarefaction over eight years is contrary to the lore that sea urchin spines that remain in human soft tissue will exhibit significant, or complete, absorption or dissolution over months to a few years.

## Introduction

When sea urchin puncture injuries occur, they often affect extremities, such as hands and feet. In a recent review of nearly 90 articles, Schwartz et al. [1] reported that 80% of sea urchin injuries affect the feet, 15% affect the hands, and the remaining 5% of injuries are distributed throughout the trunk, proximal limbs, and head. After sea urchin spines become fully embedded under the skin, various methods are used for their extraction. For example, medical professionals advocate surgical removal if simpler measures do not work and as long as secondary complications are minimized, such as causing a secondary infection or injuring a neurovascular structure during their extraction [1]. Removal or dissolution of sea urchin spines and spine fragments and reduction of local symptoms can be facilitated using various non-surgical means, including liquid nitrogen treatment, laser ablation, hot water soaks, and the application of lemon juice or vinegar (additional discussion and references appear below). Some reports recommend attempting to break down embedded spines via manual “mashing” or “crushing” at the site of injury (e.g., with the handle of a screwdriver) to facilitate breaking down the spines so that they can be absorbed [2-4]. Similar recommendations can also be found on some internet blogs and websites dealing with aquatic recreation. For example, the notion that smaller spines and spine fragments might dissolve after mashing is suggested on the internet in Wikipedia (assessed February 10, 2024):

“Embedded (sea urchin) spines can often be removed with tweezers with no long-term consequences, but their brittle nature often leaves fragments within the body [1,5,6]. Hot water soaks are thought to destroy toxins, alleviate pain, and help dissolve any remnants of the spines [1,5,6].”

We report the case of a 21-year-old male who had a sea urchin spine embedded deep in the soft tissue of the plantar aspect (sole) of his left foot after he stepped on a sea urchin while kayaking off the coast of Oahu, Hawai’i. He was told by surfers and other locals to “mash” the location of his injury with his thumb and that this and the mechanical forces of walking would accelerate the dissolution of the spine. We could not locate any reports, published or unpublished (e.g., information posted on the internet), with radiographic imaging to confirm with certainty that such treatments do lead to disintegration and ultimate significant, or complete, absorption of a deeply embedded sea urchin spine. We report this case because, to the extent of our knowledge, no other published report or available literature has described the fate of an embedded sea urchin spine that remained in a patient’s soft tissue for a minimum duration of two years (our follow-up is eight years) without infection, complications, or significant absorption. We also report this case in the context of an overview of the pertinent literature.

## Case presentation

**Figure 1:**
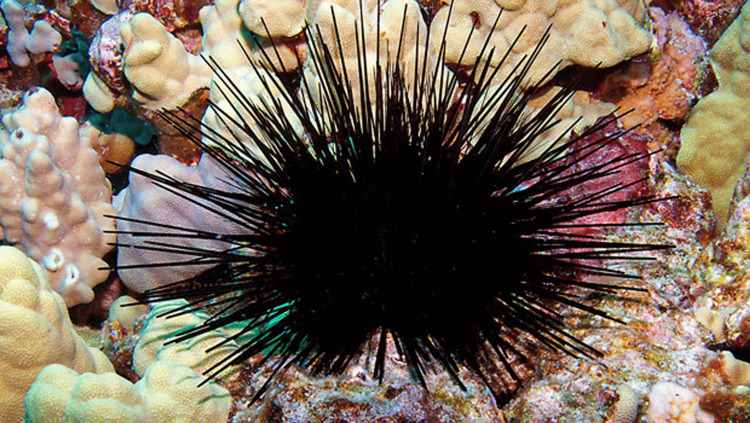
The type of sea urchin encountered by our patient Photograph of the most likely type of sea urchin (Echinothrix diadema) that our patient stepped on off the coast of Oahu, Hawai’i, USA [7,8]. Image reproduced with permission of Waikiki Aquarium, University of Hawai’i, Oahu, Hawai’i; all rights reserved [9].

On December 29, 2015, an otherwise healthy 21-year-old male was kayaking in shallow water off the coast of Oahu, Hawai’i. He was knocked over by a wave and felt a sharp pain on the plantar aspect of his bare left foot when he contacted the ocean floor. He immediately examined the sole of his left foot and found three black, needle-like spines protruding from his skin near the distal aspect of the first metatarsal. The patient looked closely at the patch of ocean floor that his foot had contacted and saw multiple black sea urchins on the ocean rocks in the shallow water (Figure 1). He was able to remove two of the three embedded spines by himself, but only a portion of the third spine came out. The patient sought care at a local urgent care facility, where the providers confirmed via plain radiographs that he still had a piece of the third spine embedded approximately one centimeter deep in the plantar sub-metatarsal tissue in the first interspace of his left foot. They instructed him to use his thumb to manually apply repetitive pressure (“mashing”) to the location of injury for several minutes daily until pain was diminished over the next few weeks.

Because of persistent pain when walking in the days following the injury, the patient presented to our clinic on January 14, 2016, in his hometown of Salt Lake City, Utah, USA. He was noted to walk with a mild limp, but there were no signs of infection at or near the site of the puncture, which had trace punctate erythema. There was mild pain with manual finger palpation over the puncture site, but no swelling. There were no limitations in the range of motion of any of this toe and mid- and hind-foot joints, and active and passive motion of his toes did not produce pain, suggesting that the spine was not in a tendon or tendon sheath [3].

**Figure 2:**
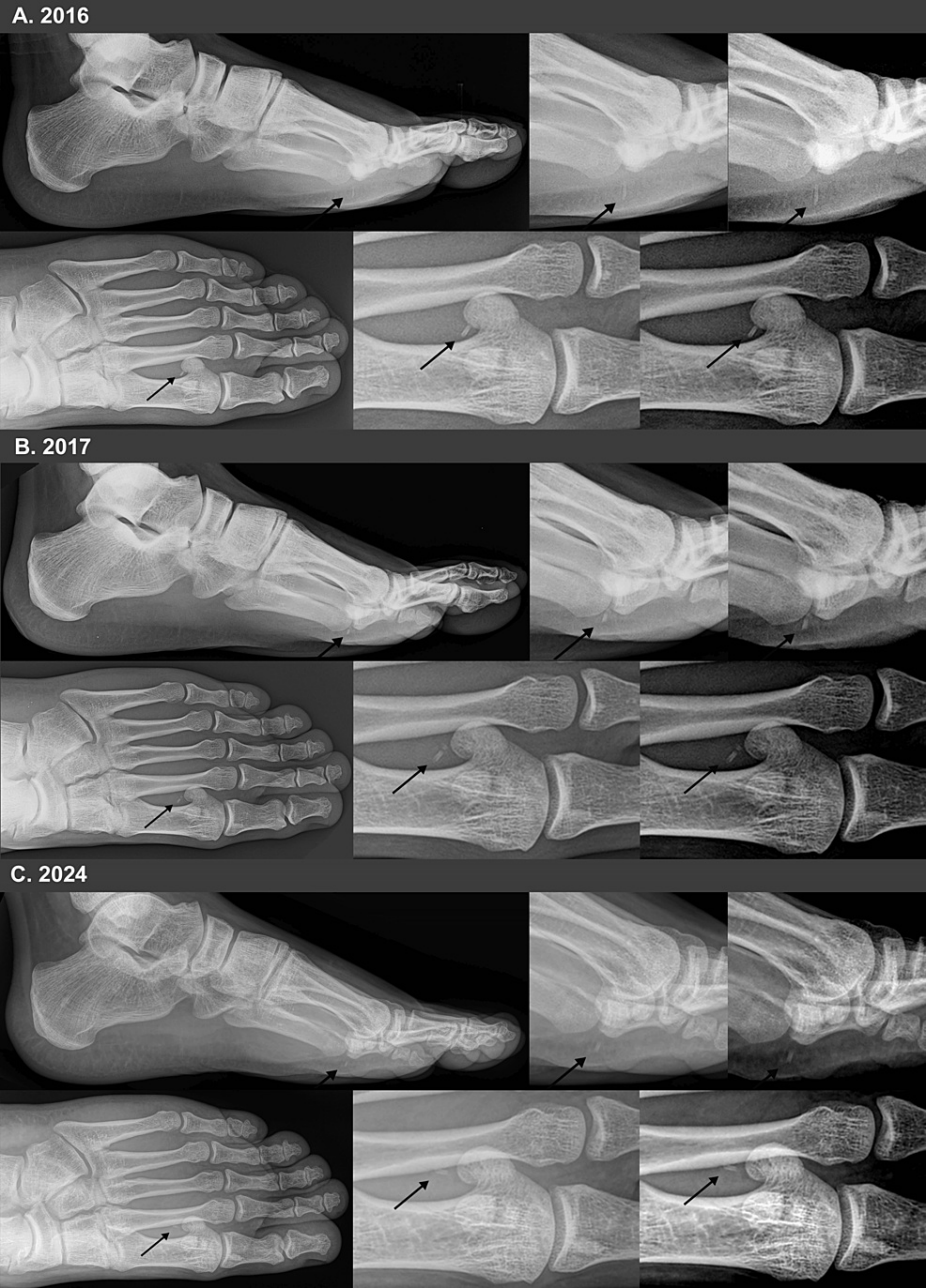
Lateral and anterior-posterior (or oblique) radiographs of the patient’s left foot A: three weeks after the injury; B: one year after the injury; and C: eight years after the injury. Arrows point to the embedded spine. In B, the spine was noted to be fragmented. Radiographs on the far right of each row have enhanced contrast in order to better visualize the sea urchin spine; all other radiographs are as seen in the clinic. The embedded spine resembles that shown in a radiograph of a 31-year-old female, where the spine was embedded in the posterior tibia tendon (and was surgically removed soon after the injury) [10].

Digital radiographs (GE Medical Systems; Siemens Model A101F; software: Merge Healthcare North America) were taken and showed the apex of a sea urchin spine measuring approximately 1.5mm x 7mm dimensions remained in the soft tissues in the plantar sub-metatarsal tissue in the first interspace of his left foot (Figure 2A). Although the differential diagnosis for this foreign body might be a fragment of coral, rock, sea shell, etc., the patient’s report that he visualized three spines protruding from his foot and that one broke and remained embedded when he attempted to extract it rendered all of these other possibilities highly unlikely. At that time, our management was based on a literature review of published cases; we had no prior experience with this specific type of injury. Given the absence of infection and the likely uncomplicated location of the spine (i.e., not in the tendon, tendon sheath, nerve, joint, or bone), the patient was advised to treat his pain with over-the-counter anti-inflammatory medication and to report any worsening or other concerning symptoms. Over the following three weeks, the patient continued to periodically mash the site with his thumb, and the pain fully subsided. He had no other symptoms thereafter.

One year later, on January 12, 2017, the patient was asked to follow up in our clinic so that he could be examined and have radiographs taken to re-evaluate the location of the urchin spine. At that follow-up appointment, he reported no pain, and there were no signs of edema, swelling, or neurovascular deficits. Radiographs showed that the sea urchin spine had fragmented into two pieces. The smaller fragment was ~15% of the prior larger single-embedded spine. Otherwise, the remaining/visible spine was of similar overall dimensions and radio-opacity as seen in January 2016 radiographs, and it was in the same location as the plantar aspect of his foot (Figure 2B).

At a 5.5-year follow-up visit (June 2021), the patient was entirely asymptomatic. Radiographs taken at that time again showed that the larger fragment of the urchin's spine had similar dimensions and radio-opacity as seen in January 2017 radiographs, and it had remained in the same location as the plantar aspect of his foot (images not shown). The smaller fragment was not visualized. However, it was unclear if it had been absorbed or was not seen because of the projection artifact. Final radiographs obtained in February 2024, just over eight years after the injury, showed that the smaller fragment was no longer visible, suggesting that it had been absorbed. Multiple radiographic projections were obtained to be sure that it was not hidden by overlapping tissue. However, the main fragment of the urchin's spine had similar dimensions and radio-opacity when compared to radiographs taken in January 2016, and the spine remained in the same location (Figure 2C). Examination of all radiographs by three independent observers (including a radiologist) with the unaided eye and with magnification showed no evidence of dissolution/rarefaction of the remaining embedded urchin spine. The same digital radiographic equipment with the capability of producing high-resolution images and the advanced capability of post-imaging contrast or brightness and magnification adjustments was used at each of the patient’s clinic visits. At the 2017, 2021, and 2024 follow-up appointments, the patient reported no pain or limitations, and physical examinations for his foot were completely normal.

## Discussion

Sea urchins are a group of benthic marine invertebrates characterized by having rigid globulous bodies that are covered in spines composed of calcite, which is a form of calcium carbonate. They belong to the phylum Echinodermata and class Echinoidea, hence their common group name "Echinoderms," which is Latinized from the Greek root words “echino” (meaning spiny) and “derma” (meaning skin). There are over 900 living species of sea urchins, but fewer than 10% are toxic to humans [11]. Sea urchins typically have spines that are stiff and brittle; therefore, they may break if they become embedded in the skin. The spines are composed of a single crystal of calcite with a crystallographic c-axis along the spine’s length [12,13]. The crystals, which are the most stable and least soluble polymorph of calcium carbonate (CaCO3), also contain magnesium and a few other elements in trace amounts [14,15]. It may be expected that a structure comprised of a single crystal of calcite would be very brittle; sea urchin spines, however, contain embedded glycoproteins within the calcite that enhance their elasticity and fracture resistance [12,16]. At the microscopic level, sea urchin spines are porous and textured [12,13,16-18]. They are covered by an epithelial layer, which is believed to contribute, in some cases, to a foreign-body inflammatory reaction if embedded beneath the epidermis of the patient [1,18-23].

Extraction methods and the notion that embedded sea urchin spines eventually absorb

There is a plethora of information on treatments for these puncture injuries, with the most common among medical professionals being the removal of all partially embedded spines and the removal of as many fully embedded spines as possible. Urban legend, local surfing cultures, and even some published literature, though limited, share the common perspective that sea urchin spines left in place in the soft tissue beneath the epidermis will gradually be extruded, dissolve, or be absorbed by the surrounding tissue [2,3,10,22,24,25]. The main finding of the persistence of the larger spine fragment in the soft tissue of our patient’s foot is not consistent with this hypothesis. The embedded sea urchin spine in our patient’s foot, with the main fragment still radiographically visible eight years after injury and of similar dimensions to the first images recorded, did not appear to have undergone significant absorption or dissolution. However, the smaller fragment had apparently been absorbed.

Chemical composition and detection in clinical imaging

Due to their chemical composition, sea urchin spines are radiopaque and visible on plain radiographs, making this the preferred technique for visualizing the presence of sea urchin material [10,26]. There are reports, however, where embedded spines were not visible on plain radiographs [5,22,24,27]. Nassab and co-workers [5] propose that a delay in the presentation of an embedded sea urchin spine to medical facilities where radiographs can be performed “may result in failure to show the spines radiographically as the surrounding tissues absorb the calcium [28].” Their reference to the case report of Newmeyer [28] is problematic. This is because the investigator reported that the embedded sea urchin spines were detected on plain or standard radiographs, though they were very faint. In cases such as these, radiographs with ‘soft tissue windows’ or with the capacity of other digital enhancements, or ultrasonography, are the next steps in the diagnostic imaging workup of sea urchin injuries [10,28-32]. Computed tomography (CT) scanning in cases of deeply embedded spines can be more useful than plain radiography and is also superior to magnetic resonance (MR) imaging for detecting embedded sea urchin spines [10].

Toxins, immediate and delayed reactions, and infections

Sea urchins may have toxins, but few species are significantly toxic to humans. Toxins are usually concentrated near their mouth, which is located on the underside of their body. Some species have toxins at the spine tips that can cause significant harm when contacted by humans. Sea urchin toxins and related substances include glycosides, hemolysins, proteases, histamines, serotonin, cholinergic substances, and bradykinin-like substances [33-35]. However, sea urchins that have toxins that are deemed significant to humans are not found along the coast of the Hawaiian Islands.

Injuries caused by the piercing of a sea urchin spine often occur in the feet or hands, followed by embedment after the brittle spine(s) break off [1,3,10,25,26]. Reactions to sea urchin puncture injuries can be classified as immediate or delayed. Immediate effects may include pain, swelling, and bleeding, followed by edema, aching, and local myalgia [1,33]. Delayed effects and possible long-term complications usually depend on whether the spines penetrate tendons, tendon sheaths, bones, or joints and/or become infected. Complications may include arthritis, granulomas, impaired function, synovitis, persistent neuropathy and tendinopathy, chronic arthropathy, and delayed hypersensitivity reactions [3,22,26,33,36-40]. Additionally, secondary infections and tetanus may manifest due to the presence of a lodged foreign body [3,41]. Fortunately, our patient did not experience any delayed reactions or other complications.

Fibrous encapsulation and the unlikely/minimal dissolution of calcite in many cases

One of the three anhydrous calcium carbonate crystalline phases is calcite, which comprises sea urchin spines [15,42,43]. Because body fluids are supersaturated relative to calcium carbonates, spontaneous dissolution in vivo is unlikely [44], casting doubt on the possibility that significant passive absorption of sea urchin spine would occur over a timeframe of several weeks to months. Additionally, passive dissolution of sea urchin calcite also seems unlikely from the perspective that it is the most stable and least soluble polymorph of calcium carbonate (CaCO3) [15,43]. While it might be expected that enzymatic or other cell-mediated processes would degrade a sea urchin spine embedded in soft tissue, it is suggested that this might not occur because the spine would be expected to become rapidly encapsulated by fibrous tissue, which is a foreign body response [45]. It is known that when organic foreign bodies are embedded within soft tissues, fibrous encapsulation typically occurs [46,47]. Before the fibrous encapsulation of a sea urchin spine embedded into soft tissue, the outer epithelial layer of the spine can trigger an immune reaction [19,48]. However because this layer is very thin and mostly proteinaceous, a significant immune reaction would be expected to last for a few days to a week [48]. Fibrous encapsulation greatly reduces, and can eventually eliminate, blood flow in the vicinity of the foreign body, excluding the sea urchin spine from the active phagocytic and degradative processes of the immune system (e.g., macrophages, etc.) [46]. As discussed below, this is one of the reasons that relatively larger foreign bodies made of absorbable/resorbable material are usually not completely resorbed. Nevertheless, very minor surface dissolution of the larger embedded spine fragment in our patient might have occurred but was undetectable to the unaided eye. Perhaps detectable breakdown/dissolution of the larger spine fragment embedded in our patient’s foot requires more than eight years. Evidence from case reports suggests that breakdown can occur within months after embedment if the local inflammatory reaction is more substantial, which likely depends on the location of embedment; for example, within a joint where more mechanical perturbation can occur, evoking synovitis/arthritis and subsequent cellular recognition and degradation [22,29].

Observations of bioabsorption of calcium carbonate in coral (aragonite) vs. calcite of sea urchins

The idea that an embedded sea urchin spine might be absorbed in the subcutaneous, fascial, or muscular tissues of the human body has some support in observations made when calcific biominerals of other marine organisms are embedded in the skin, fibrous, or muscular tissues of non-marine mammals. These observations have been made in rats when used as experimental animals for human orthopedic applications [49-53]. For example, in the late 1970s, coral began to be successfully used in humans as a bone graft material [53,54]. The calcium carbonate polymorph found in sea urchins is very similar to the calcium carbonate polymorph known as aragonite that is found in the exoskeletons of nearly all coral species [42,43,53]. Naturally occurring aragonite is known to be osteoconductive and bioresorbable [50,52,54]. Ohgushi et al. [50] showed that marine coral calcium-carbonate implants (i.e., aragonite) embedded in the subcutaneous tissue of rats underwent more phagocytic activity than implants made of hydroxyapatite (HA), which is a natural biomineral of mammalian bone. The “implants” that they used were discs that were 5mm in diameter and 2mm thick with 50-60% fully interconnected porosity. However, the fate of the implants was followed for only eight weeks, and they were partially absorbed. In another study, Nishikawa and co-workers [51] studied the fates of three different porous calcium-containing particles in the dorsal subcutaneous tissue of rats: (1) two calcium phosphate particle sizes: 10um Ca3(PO4)2, 30um Ca3(PO4)2 (neither found in sea urchin spines), and (2) 60um CaCO3. It is worth noting that the authors did not provide sufficient information to determine whether or not the calcium carbonate that was used for the 60um particles is the same polymorph found in sea urchin spines [43]. Nevertheless, all the phosphate-containing particles and a large majority of the calcium carbonate (CaCO3) particles were completely absorbed after eight weeks. In these experimental studies, absorption of calcium carbonate polymorphs was facilitated by the phagocytic activity of foreign-body giant cells (which are a special type of macrophage) when embedded in subcutaneous tissue [50,51,55].

The fact that Nishikawa and co-workers [51] reported near-complete absorption of calcium carbonate particles is likely due, in part, to the fact that these particles were tiny, < 60um in diameter. The much larger size of the sea urchin spine embedded in our patient’s foot likely exceeds the phagocytic capabilities of macrophages, particularly foreign-body giant cells. This idea is supported by observations in studies of wear-debris-related particle disease associated with total hip replacement implants. These studies have reported that microscopic particles of metal, plastic, or bone cement that are produced from the wear of the implants are much more likely to be ingested by macrophages and evoke an immune reaction resulting in bone and implant loosening. In contrast, larger debris particles and fragments are seemingly inert, where neither phagocytosis nor immune reaction occurs [56,57].

The standard of care is the removal

Early surgical intervention is suggested for embedded sea urchin spines and is the standard of care for those embedded in joints, tendons, tendon sheaths, and neurovascular structures to prevent delayed reactions [1]. A comprehensive treatment algorithm can be found in Schwartz et al. [1]. They recommend against the mashing/crushing of spines in situ with the intent of inducing rapid absorption because they suggest that there is evidence that this intervention can push the spines deeper into underlying structures [2,3]. Most treatments that we have reviewed in the published and unpublished literature involve the immediate removal of visible sea urchin spines after injury. However, the brittle nature of sea urchin spines makes complete removal difficult in many cases, and attempts to remove spines can secondarily cause an infection [25]. As shown by our patient’s case, a sea urchin spine that is not embedded near-critical regions might not require surgical intervention. We suspect that in many cases, because symptoms from one or a few embedded sea urchin spine tips improve rather quickly, they are not removed and are ultimately deemed innocuous [31,58]. Similarly, in our patient, despite the presence of an embedded spine eight years after injury, he reported no pain or discomfort.

Nevertheless, we recommend that healthcare providers follow the treatment algorithm of Schwartz et al. [1] when a patient presents with an embedded sea urchin spine. This is important because embedded spines might eventually become painful and associated with nodules, including the late development of foreign-body-type granulomas. Examples of such delayed presentations after the embedment of sea urchin spines include: (1) the recognition of painful nodules five weeks later after embedment into the skin of the thigh of a 29-year-old healthy male [37]; (2) two years after embedment into the dorsa of the hands of a 49-year-old healthy male [59]; (3) three months after embedment into the Achilles tendon of a 43-year-old male [60]; (4) 10 years after embedment into the flexor tendon sheath of a finger in a 21-year-old healthy female who was 11 years old at the time of injury [39]; and (5) 10 months after embedment into the interphalangeal joint of the hallux of a 33-year-old female [22]. Notably, none of these reports or any others that we examined showed embedded urchin spines in early radiographs and their subsequent disappearance in later radiographs in cases where delayed synovitis/arthritis, erosive inflammation, or granulomas did not occur. However, in nearly all of these cases, radiographs were often not obtained over long periods after injury. Various methods to treat such injuries are listed in Table 1.

**Table 1:** Selected cases of embedded sea urchin spines and subsequent treatments * “Local measures” include hot packs, hot water soaks, vinegar soaks, mashing, etc.

Reference	Gender, age (years)		Location	Symptoms	Treatment
O'Neal et al. [61]	Male, 30	Right-hand		Swelling and discoloration	Administration of salicylates, local heat, followed by complete removal
Cracchiolo and Goldberg [62]	Male, 45	Heel		Heel ulcer, granuloma, systemic illness, joint pain, malaise	Excision of spines and granuloma, corticosteroids
Cracchiolo and Goldberg [62]	Male, 54	Hand		Hand and shoulder pain, swelling, malaise	Prednisone, phenylbutazone
Cracchiolo and Goldberg [62]	Female, 34	Heel		Heel ulcer granuloma	Excision of spines and granuloma
Cracchiolo and Goldberg [62]	Male, 30	Knee		Massive knee synovitis	Aspiration and injection of the knee; arthrotomy, excision of spine
Cracchiolo and Goldberg [62]	Male, 27	Ankle		Ankle synovitis	Excision of spines
Cracchiolo and Goldberg [62]	Female, 25	Foot		Pain and swelling of hallux metatarsophalangeal joint	Local measures*, excision of spines
Cracchiolo and Goldberg [62]	Female, 53	Foot		Pain and swelling only, secondary to infection	Partial removal of spines, antibiotics, local measures *
Cracchiolo and Goldberg [62]	Female, 30	Foot and hand		Pain and swelling of interphalangeal joint	Partial removal of spines, local measures*
Cracchiolo and Goldberg [62]	Male, 50	Hand		Local pain and swelling of thumb metacarpophalangeal joint	Local measures*
Falkenberg [2]	Female, 36	Foot		Minimal pain following immediate in situ treatment	In situ "crushing" with rock and urine treatment
Newmeyer III [28]	Female, 43	Left-hand		Swelling, stiffness	Complete removal
McWilliam et al. [63]	Not reported	Finger		Local acute inflammatory reaction with microabscess formation	Complete removal
Morris et al. [39]	Female, 10	Left-hand		Pain and swelling of the left hand, 10-year history of chronic pain and stiffness affecting index finger	Antibiotics initially, tenosynovectomy of the flexor digitorum profundus tendon 10 years later for chronic tenosynovitis
Liram et al. [64]	Male, 42	Left-hand		Pain, stiffness, and swelling of interphalangeal joint	Ultrasound therapy, hot packs, and mobilization
Rossetto et al. [59]	Male, 49	Left-hand		Pain, stiffness, and swelling near dorsum right proximal index finger	Complete removal
Sharma et al. [25]	Female, 49	Left-foot		Not reported	Initial treatment with “green paw-paw poultice” and later destruction with Erbium-YAG laser treatment
Dahl et al. [26]	Female, 55	Right-hand		Stiffness and diminished sensation to right ring finger	Complete removal
Gargus and Morohashi [65]	Male, 23	Foot		Pain while walking	Partial removal followed by treatment with liquid nitrogen and manually extracting residual spines once epidermis blisters
Gungor et al. [66]	Female, 22	Right and left feet		Pain in both feet	Laser ablation
Hafez and Al-Dars [36]	Male, 36	Foot		Local infection and blisters	Complete removal
Schefflein et al. [22]	Female, 33	Left hallux		Pain, swelling, limited ROM, granulomatous synovitis	Complete removal and antibiotics; at 10 months post-injury synovectomy, debridement, and arthrodesis of the IP joint for severe erosive arthropathy
Mahon et al. [67]	Male, 50	Right middle finger		Pain, swelling, finger stiffness, arthritis	Oral and IV antibiotics, limited synovectomy
Hseih et al. [37]	Male, 29	Right thigh		Intermittent pain, granulomas	Complete removal
James et al. [31]	Male, 32	Left lateral foot		Pain, swelling	No excision; improved with time
Present study	Male, 21	Left-foot		Pain	No removal

## Conclusions

In our young adult male patient, a sea urchin injury resulted in an embedded spine in soft tissue in the sole of his left forefoot. An eight-year follow-up with digital high-resolution radiographic imaging showed that the majority of the spine remained embedded without any visible signs of migration or absorption. In this case, surgical removal was not necessary, and our patient has experienced a complete recovery from this injury. Our observations do not support the idea that a sea urchin spine embedded in soft tissue will eventually be absorbed, at least over an eight-year period. Because we have described only one patient, additional reports/cases are needed to determine if our findings are generally applicable in similar, uncomplicated cases.
